# 

**Published:** 1867-10

**Authors:** 


### Books and Pamphlets Received.

A Biennial Retrospect of Medicine, Surgery, and their Allied Sciences. Edited by Mr. H. Power, Dr. Anstie, Mr. Holmes, Mr. Thomas Windsor, Dr. Barnes, and Dr. C. Hilton Fagge, for the New Sydenham Society. Philadelphia: Lindsay & Blakiston, 1867. For sale by Theodore Butler.

Epidemic Meningitis, or Cerebro-Spinal Meningitis. By Alfred Stillé, M. D., Professor of the Theory and Practice of Medicine and of Clinical Medicine in the University of Pennsylvania, etc. Philadelphia: Lindsay & Blakiston, 1867. For sale by Theodore Butler.

Circular No. 7, War Department, Surgeon-General's Office, Washington, June 1, 1867. A Report on Amputations at the Hip-Joint in Military Surgery. Washington, 1867.

The Transactions of the American Medical Association, instituted 1847. Vol. 17. Philadelphia: Printed for the Association, 1867. For sale by Theo. Butler.

Synopsis of the Course of Lectures on Materia Medica and Pharmacy, delivered in the University of Pennsylvania, with five Lectures on the Modus Operandi of Medicines. By Joseph Carson, M. D. Fourth edition, revised. Philadelphia: Henry C. Lea, 1867. For sale by Breed, Lent & Co.

Studies in Pathology and Therapeutics. By Samuel Henry Dickson, M. D., LL.D., Professor of Practice of Physic in Jefferson Medical College, Philadelphia, etc. New York: Wm. Wood & Co., 61 Walker st., 1867. For sale by Breed, Lent & Co.

Hufeland's Act of Prolonging Life. Edited by Erasmus Wilson, F. R. S., author of "A System of Human Anatomy," "Diseases of the Skin," etc. Philadelphia: Lindsay & Blakiston, 1867. For sale by Theodore Butler.

Transactions of the Twenty-second Annual Meeting of the Ohio State Medical Society, held at Yellow Springs, Ohio, June 11th and 12th. 1867.

A Contribution to the History of the Hip-Joint Operation. performed during the late Civil War, being the Statistics of Twenty Cases of Amputation and Thirteen of Resections, at the Articulation, in the Southern service. By Paul Eve, M. D., Professor of Surgery in the University of Nashville, Tenn., extracted from the Transactions of the American Medical Association.

